# Perception of Weight and Health Status among Women Working at Health Centres of Tehran

**Published:** 2014-03

**Authors:** Ahmad Reza Dorosty, Sepideh Mehdikhani, Gity Sotoudeh, Abbas Rahimi, Fariba Koohdani, Parastoo Tehrani

**Affiliations:** ^1^National Nutrition and Food Technology Research Institute, Shahid Beheshti University of Medical Sciences, Tehran, Iran and Department of Community Nutrition, School of Nutritional Sciences and Dietetics, Tehran University of Medical Sciences, Tehran, Iran; ^2^Department of Community Nutrition, School of Nutritional Sciences and Dietetics, Tehran University of Medical Sciences, Tehran, Iran; ^3^Department of Epidemiology and Biostatistics, School of Public Health, Tehran University of Medical Sciences, Tehran, Iran; ^4^Department of Cellular, Molecular Nutrition, School of Nutritional Sciences and Dietetics, Tehran University of Medical Sciences, Tehran, Iran; ^5^Department of Social Medicine, School of Medicine, Tehran University of Medical Sciences, Tehran, Iran

**Keywords:** Body mass index, Health centres, Perception of health status, Perception of weight status, Waist-to-hip ratio, Iran

## Abstract

Perception of body-weight status is an important determinant of weight-related behaviours and may affect the burden of weight disturbances as a public-health problem. No study has assessed self-perception of the weight status regarding body-fat distribution among health workers to date. The aim of this study was to evaluate the association of the perception of weight and health status among 542 women working at health centres of Tehran. We assessed their perceived body-weight and health status and measured waist- and hip-circumference, weight, and height to calculate waist-to-hip ratio (WHR) as a measure of fat distribution and body mass index (BMI, kg/m^2^). Women reported their sociodemographic information, and the perceived weight and health status were compared with their actual fatness status, defined based on WHR and BMI, to determine misperception of weight status. Multivariate logistic regression models were performed to assess the predictive effects of various sociodemographic factors and actual fatness on the perception of weight and health status. The results showed that more than 40% of women with normal BMI overestimated their body-weight status while only 15.8% of these women had central obesity. BMI was the most important variable associated with misperceived weight status as normal-weight women had significantly more misperception (OR 8.16, 95% CI 4.82-13.82) than overweight/obese women. WHR did not show any significant relationships with perceived weight status. In addition, perception of health status was not associated with actual fatness indices. It is concluded, BMI was the main predictor of the perception of weight status in female employees. The importance of using body-fat distribution in the perceptions of weight and health status should be emphasized.

## INTRODUCTION

Overweight and obesity are well-known risk factors of cardiovascular diseases, diabetes, some cancers, and sleep apnea (1). The prevalence of obesity is higher in women than men (2). The results of the First National Surveillance of Risk Factors of Non-communicable Diseases (SuRFNCD) in Iran showed that about 48% of adult women were overweight or obese according to their body mass index (BMI). In addition, abdominal obesity was present in 43.4% of women (3). These figures are evidence of the high prevalence of overnutrition among Iranian females. Perception of body-weight status plays an important role in weight management (4), and underestimation of body-weight can be a risk factor for obesity in some people (5). On the other hand, overestimation of the weight by underweight or normal weight subjects can be a risk factor for unhealthy weight control practices (6) and may cause eating disorders (7). Therefore, misperception of weight may have adverse effects on nutritional behaviours. Understanding incorrect perception of weight status is important for the prevention of depression, social anxiety, and eating disorders (8). Despite the importance of the perception of weight, only a few studies have assessed perception of body in adolescents (9-11) and adult women (12) in our country.

BMI has been used as the standard reference for comparing perception of weight status with actual adiposity status in many studies (13,14). Although body-fat distribution, which is not measured by BMI, may affect perception of weight status as well, this issue has been investigated very rarely (15,16). Waist-to-hip ratio (WHR), as an additional measure of body-fat distribution, is a stronger risk factor compared to BMI for myocardial infarction, stroke, and premature death in women (17).

Health centres are the most important settings for health education and promotion. Health workers have a significant role in providing services, including nutritional education to women who attend these centres. Though not investigated, it is reasonable to assume that perception of health workers of their weight and health status may affect the perception of attendants. We are not aware of any study that has evaluated perception of weight status among health workers. Therefore, the aim of this study was to determine perception of weight and health status among female health workers in urban health centres in Tehran and to examine their association with actual fatness and BMI, using WHR as the most reliable measure.

## MATERIALS AND METHODS

### Subjects

This cross-sectional study was conducted in 2010 at urban health centres in Tehran. In this city, all health centres are affiliated to two main medical universities (Tehran University and Shahid Beheshti University). A single-stage stratified cluster-sampling method was used for selecting participants. For sampling, the list of all health centres (n=325) was prepared as the sampling frame. These centres were stratified by the two universities. Simple random sampling was employed with probability proportional to the size of each stratum. Forty-three centres affiliated to Tehran University and 73 centres affiliated to Shahid Beheshti University were selected. Each centre was considered a cluster, and all women who were working at each selected centre were invited to participate in the study. Estimation of the sample-size was based on the percentage of misperception of weight status, which was reported as 48% in a study on Iranian women (12). The sample-size was determined with a confidence level of 95%, using p=0.48, d=0.09×0.48=0.043, α=0.05 as per the equation n=Z^2^P(1-P)/d^2^ where Z=1.96, P=0.48, and d=0.043. Accordingly, our sample-size was determined to include 532 participants. In practice, 562 women were interviewed. Pregnant and/or lactating women were not included and, due to the small sample-size, underweight females (BMI <18.5, n=20) were excluded from the study. As a result, a total of 542 female health workers were studied.

The study protocol was approved by Medical Ethics Committee of Research Deputy for Shahid Beheshti University of Medical Sciences.

A questionnaire containing the sociodemographic data was completed in the first step. Variables included age (35 or less and more than 35 years), education as the prosperous years of schooling (12 or less and more than 12 years), years of service (10 or less and more than 10 years), marital status (single, married, and widowed or divorced), job (technician/expert of health, nutrition, or midwifery; physician, dentist, or pharmacist; employee and manual worker), and the present known disease (yes and no).

The perception of individuals about their body-weight status was assessed using the following question (which has been used in other studies as well) (15,18,19): “Do you imagine yourself as underweight, normal, overweight, or obese?”

To determine the perception of body health status, the participants were asked if they imagined their health status as excellent, good, average, poor, or very poor. The responses were then categorized in three levels of excellent/good, moderate, poor/very poor. When health status was assessed as a dependent variable, the responses were classified into 2 categories: excellent/good and moderate/poor/very poor.

### Anthropometric measurements

Weight and height were measured with minimal clothing and without shoes. Weight was measured to the nearest 0.1 kg, using a weighing scale (Seca Model 700, Germany), which was calibrated on a daily basis with known weights. In order to correct the weight of the clothing, one kg was deducted from all obtained weights. This estimate was based on the mean weight of clothing in 20 women. Waist-circumference was measured at the level of the midway between the lowest rib and the iliac crest while hip-circumference was measured at the level of the great trochanter. BMI was calculated as weight (kg) divided by the square of the height (in metre). WHR, as a measure of body-fat distribution, was calculated by dividing waist-circumference (cm) by hip-circumference (cm).

Actual fatness status of the participants was determined in three ways: (i) normal fatness (WHR <0.85), or centrally obese (WHR ≥0.85); (ii) normal weight (BMI 18.5-24.9); overweight (BMI 25-29.9), or obese (BMI ≥30); (iii) considering both indices of WHR and BMI: a. normal WHR and BMI, b. centrally obese and normal BMI, c. normal WHR and BMI-based overweight/obese, and d. centrally obese and BMI-based overweight/obese.

The participants’ responses to perceptions of their body-weight status were compared with their actual fatness status to determine misperception of weight status. For women who had central obesity, regardless of their BMI levels, misperception was considered when the response was ‘underweight’ or ‘normal weight’. To assess the misperception of weight status in women with a normal WHR, their BMI levels were considered as well. WHR may be a much better gold standard for healthy weight than BMI. Since some women with a normal WHR may have a high BMI, which is also associated with an increased risk of chronic diseases (17), we used both indices for misperception of the definition of weight in women who had a normal WHR. When BMI was assessed as an independent variable for perception of weight and health status, their levels were classified into 2 categories: normal weight and overweight/obese.

### Statistical analysis

Statistical analyses were performed using SPSS 11.5 for Windows (SPSS Inc., Chicago, IL, USA). Crude odds ratios with 95% confidence intervals were calculated to analyze associations among perception of weight and health status and anthropometric and sociodemographic variables. Multivariate analyses were conducted using a logistic regression model to assess the potential importance of various variables. All tests for statistical significance were two-tailed and were performed assuming a type I error probability of <0.05.

## RESULTS

### Characteristics of the study population

The mean age of the participants was 37.3 years (SD±8.9). About 28% of the women had central obesity. Overweight and obesity according to BMI was present in 31.4% and 15.1% of the participants respectively. Twenty-one percent of the women had both central obesity and overweight/obesity (Table 1).

**Table 1.** Characteristics of participants

Characteristics	n (%)
Age (years)	
≤35	158 (29.2)
>35	384 (70.8)
Education (years of schooling)	
≤12	94 (17.3)
>12	448 (82.7)
Years of service	
≤10	211 (38.9)
>10	331 (61.1)
Marital status	
Single	136 (25.1)
Married	377 (69.6)
Widowed/divorced	29 (5.4)
Job	
Technician/expert of health, nutrition, or midwifery	349 (64.4)
Physician, dentist, or pharmacist	58 (10.7)
Employee	100 (18.5)
Manual worker	35 (6.5)
Present known disease	
Yes	165 (30.4)
No	377 (69.6)
WHR	
Normal fatness (<0.85)	390 (72)
Central obesity (≥0.85)	152 (28)
BMI (kg/m^2^)	
Normal (18.5-24.9)	290 (53.5)
Overweight (25.0-29.9)	170 (31.4)
Obese (≥30.0)	82 (15.1)
Combined index (WHR and BMI)	
WHR <0.85; BMI 18.5-24.9	252 (46.5)
WHR ≥0.85; BMI 18.5-24.9	38 (7.0)
WHR <0.85; BMI ≥25.0	138 (25.5)
WHR ≥0.85; BMI ≥25.0	114 (21.0)

BMI=Body mass index;

WHR=Waist-to-hip ratio

### Association of the perception of weight status with actual fatness

Tabulating perception of weight status with actual fatness showed that most of the women had a correct impression of their adiposity status. However, more than 40% of women with a normal BMI imagined themselves to be overweight or obese. The women with a normal or high BMI, who had central obesity, reported perceptions of higher overweight or obesity. However, about 47% of those with a normal weight status according to BMI, who had simultaneous central obesity, thought their weight status was normal (Table 2).

Bivariate analyses showed that the odds ratios of the misperception of weight status were significantly higher among the younger, those with less years of service, and those who had a normal WHR or BMI (Table 3). Adjusted analyses showed that the women with 12 or less years of education had significantly higher odds of misperception than their counterparts. BMI was the most important variable associated with misperception of weight status as women with normal weight had significantly more misperception than overweight/obese women (OR 8.16, 95% CI 4.82-13.82).

### Association of the perception of health status with actual fatness

Tabulating perception of health status with actual fatness showed that 12.5% of the centrally-obese women and 10.3% of those with overweight or obesity according to BMI had poor/very poor perception of their health status. This figure was 14% in those with high levels of both WHR and BMI (Table 4).

**Table 2.** Distribution of women's perceptions of weight status and actual fatness

Actual fatness	Perception of weight status		
	Underweight No. (%)	Normal weight No. (%)	Overweight/Obese No. (%)
WHR		
Normal fatness (<0.85)	14 (3.6)	153 (39.2)	223 (57.2)
Central obesity (≥0.85)	1 (0.7)	29 (19.1)	122 (80.3)
BMI (kg/m^2^)		
Normal weight (18.5-24.9)	15 (5.2)	155 (53.4)	120 (41.4)
Overweight (25.0-29.9)	0	26 (15.3)	144 (84.7)
Obese (≥30.0)	0	1 (1.2)	81 (98.8)
Combined index (WHR and BMI)		
WHR <0.85; BMI 18.5-24.9	14 (5.6)	137 (54.4)	101 (40.1)
WHR ≥0.85; BMI 18.5-24.9	1 (2.6)	18 (47.4)	19 (50.0)
WHR <0.85; BMI ≥25.0	0	16 (11.6)	122 (88.4)
WHR ≥0.85; BMI ≥25.0	0	11 (9.6)	103 (90.4)

BMI=Body mass index;

WHR=Waist-to-hip ratio

Bivariate analyses showed that the odds ratios of perceived moderate/poor/very poor health status were significantly lower among the young, those with less years of service, single or married, those who were technician/expert of health, nutrition, midwifery, physician, dentist or pharmacist, and had a normal WHR or BMI. Women who were less educated or had known diseases showed higher odds of perceived moderate/poor/very poor health status (Table 5). After adjusting for WHR, BMI, and sociodemographic variables, these associations remained significant only for education (OR 2.02, 95% CI 1.01-4.03) and known disease (OR 2.92, 95% CI 1.95-4.37).

## DISCUSSION

In this study, women's perception of their body-weight and health status was evaluated and its association with actual fatness was examined, using both WHR and BMI indices. The result of the present study showed that improper perception, especially the preference for slimness, was present in many female employees working at the health centres in Tehran. BMI was the strongest predictor of the misperception of weight status as normal-weight women were more likely to misperceive their weight status compared to overweight/obese women. WHR, as a measure of body-fat distribution, did not affect women's perceptions of weight status. In addition, perception of health status was not related to fatness status.

**Table 3.** Association between women's misperception of weight status and sociodemographic characteristics, BMI, and WHR

Variable	Misperception of weight No. (%)	Unadjusted OR (95% CI)	p value	Adjusted* OR (95% CI)	p value
Age (years)					
≤35	82 (36.1)	1.68 (1.16-2.45)	0.006	0.83 (0.43-1.59)	0.5
>35	79 (25.1)	1.0		1.0	
Education (years of schooling)					
≤12	33 (35.1)	1.35 (0.84-2.16)	0.2	2.24 (1.04-4.80)	0.03
>12	128 (28.6)	1.0		1.0	
Years of service					
≤10	81 (38.4)	1.95 (1.34-2.84)	<0.001	1.60 (0.85-3.02)	0.1
>10	80 (24.2)	1.0		1.0	
Marital status					
Single	47 (34.6)	1.17 (0.49-2.78)	0.7	0.9 (0.32-2.53)	0.8
Married	105 (27.9)	0.85 (0.37-1.94)	0.7	1.23 (0.47-3.20)	0.6
Widowed/divorced	9 (31.0)	1.0		1.0	
Job					
Technician/expert of health, nutrition, or midwifery	103 (29.5)	0.80 (0.38-1.67)	0.5	0.75 (0.24-2.33)	0.6
Physician, dentist, or pharmacist	12 (20.7)	0.50 (0.19-1.28)	0.1	0.49 (0.13-1.87)	0.3
Employee	34 (34.0)	0.98 (0.43-2.22)	0.9	0.86 (0.30-2.47)	0.7
Manual worker	12 (34.3)	1.0		1.0	
Present known disease					
Yes	46 (27.9)	0.88 (0.58-1.32)	0.5	1.06 (0.67-1.69)	0.7
No	115 (30.5)	1.0		1.0	
WHR					
Normal fatness (<0.85)	131 (33.6)	2.05 (1.31-3.23)	0.002	1.23 (0.70-2.16)	0.4
Central obesity (≥0.85)	30 (19.7)	1.0		1.0	
BMI (kg/m^2^)					
Normal weight (18.5-24.9)	134 (46.2)	7.15 (4.51-11.35)	<0.001	8.16 (4.82-13.82)	<0.001
Overweight/Obese (≥25.0)	27 (10.7)	1.0		1.0	

*Adjusted for age, education level, years of service, marital status, job, present known disease, BMI, and WHR;

BMI=Body mass index;

CI=Confidence interval;

OR=Odds ratio;

WHR=Waist-to-hip ratio

The odds of misperception of weight status were more than 8 times higher in women with normal weight, based on BMI, than overweight/obese subjects, which is probably due to dissatisfaction on body-weight status among these women. Central obesity was present only in 15.8% (n=19) of the women with normal weight, who considered themselves as overweight/obese. Therefore, the majority of subjects with normal weight overestimated their adiposity status. Other studies have also confirmed that non-obese women are more probable to have an inaccurate perception of weight status when compared with obese women (20). The relationship between perception of weight status and BMI is also reported in other studies (14,21,22). The odds of perceived heavier weight status in women and men increased by 60% for each unit increase in BMI (14).

**Table 4.** Distribution of women's perceptions of health status, actual fatness, and weight status

Actual fatness	Perception of health status		
	Excellent/good (%)	Moderate (%)	Poor/very poor (%)
WHR			
Normal fatness (<0.85)	218 (55.9)	147 (37.7)	25 (6.4)
Central obesity (≥0.85)	65 (42.8)	68 (44.7)	19 (12.5)
BMI (kg/m^2^)			
Normal (18.5-24.9)	165 (56.9)	107 (36.9)	18 (6.2)
Overweight (25.0-29.9)	87 (51.2)	67 (39.4)	16 (9.4)
Obese (≥30.0)	31 (37.8)	41 (50.0)	10 (12.2)
Combined index (WHR and BMI)			
WHR <0.85; BMI 18.5-24.9	148 (58.7)	89 (35.3)	15 (6.0)
WHR ≥0.85; BMI 18.5-24.9	17 (44.7)	18 (47.4)	3 (7.9)
WHR <0.85; BMI ≥25.0	70 (50.7)	58 (42.0)	10 (7.2)
WHR ≥0.85; BMI ≥ 25.0	48 (42.1)	50 (43.9)	16 (14.0)
Perception of weight			
Underweight	4 (26.7)	9 (60.0)	2 (13.3)
Normal weight	108 (59.3)	60 (33.0)	14 (7.7)
Overweight/Obese	171 (49.6)	146 (42.3)	28 (8.1)

BMI=Body mass index;

WHR=Waist-to-hip ratio

Consistent with previous studies (13,23), we found that education was related to misperception of weight status. The women with lower levels of education had significantly higher odds of misperception than their counterparts.

In univariate analysis, WHR showed a statistically significant association with misperception. However, after adjusting for other variables, this association was not statistically significant anymore. This finding reveals that women's perception of weight status, instead of WHR, is mainly based on their BMI. WHR has been recognized as a better marker for assessing health status and mortality risk than BMI (24,25). BMI is commonly used for classification of overweight and obesity. However, it has some limitations which could lead to misclassification of fatness status in individuals (26,27). BMI does not differentiate between body-fat and muscle mass. There is a potential risk of overestimating ‘fatness’ in individuals with high muscle mass or bone density, such as body-builders and manual labourers.

Most of the women with central obesity in this study properly perceived their fatness status. However, women with a high BMI were more likely to perceive themselves overweight or obese in comparison with women who had a normal BMI and high WHR, which is also shown in a study on Canadian women (15). A possible explanation may be that, in comparison with men, fat storage in women is mostly concentrated in the gluteal-femoral region; as a result, they may have a more general awareness of their body-weight status. Therefore, their judgement for adiposity may be mainly based on BMI, and they may pay less attention to fat storage in the mid-section of the body (28). However, subjects with central obesity are at a higher risk even in the absence of a high BMI. The inappropriate perception in these people prevents them from taking action in order to decrease central obesity.

Data analysis showed that 46.5% of women were suffering from being overweight or obese, which is consistent with the obesity/overweight rate among women in the country. However, the prevalence of central obesity among the study population was lower than the rate in the country. Generally, about one-third of the study population had inaccurate perception of their adiposity. Regarding central obesity, 5.5% of women did not consider themselves as overweight/obese. Based on BMI, underestimation of weight status was present in 3% and overestimation in 41.4% of the women. Underestimation and overestimation of body fatness, based on BMI, are reported to be present respectively in 34.5% and 13.5% of women in Islamshahr (12), which is far different from the results of the present study. These rates are reported to be 12.2% and 17.7% in Malaysian employees (20) and 3.9% and 42.4% in Japanese female employees (29) respectively, showing that overestimation of weight status, based on BMI, in our study population was similar to that of Malaysian employees but much less than Japanese female employees.

**Table 5.** Association between women's perceptions of moderate/poor/very poor health status and sociodemographic characteristics, BMI, and WHR

Variable	Perception of health status* No. (%)	Unadjusted OR (95% CI)	p value	Adjusted† OR (95% CI)	p value
Age (years)					
≤35	89 (39.2)	0.55 (0.38-0.77)	0.001	1.15 (0.65-2.02)	0.6
>35	170 (54.0)	1.0		1.0	
Education (years of schooling)					
≤12	62 (66.0)	2.46 (1.55-3.93)	<0.001	2.02 (1.01-4.03)	0.04
>12	197 (44.0)	1.0		1.0	
Years of service					
≤10	78 (37.0)	0.48 (0.34-0.69)	<0.001	0.59 (0.34-1.04)	0.06
>10	181 (54.7)	1.0		1.0	
Marital status					
Single	54 (39.7)	0.29 (0.12-0.69)	0.005	0.52 (0.20-1.38)	0.1
Married	185 (49.1)	0.43 (0.19-0.97)	0.04	0.56 (0.23-1.37)	0.2
Widowed/divorced	20 (69.0)	1.0		1.0	
Job					
Technician/expert of health, nutrition or midwifery	157 (45.0)	0.37 (0.17-0.78)	0.01	1.12 (0.39-3.24)	0.8
Physician, dentist, or pharmacist	24 (41.4)	0.32 (0.13-0.78)	0.01	0.90 (0.27-2.93)	0.8
Employee	54 (54.0)	0.53 (0.23-1.21)	0.1	1.05 (0.39-2.78)	0.9
Manual worker	24 (68.6)	1.0		1.0	
Present known disease					
Yes	111 (67.3)	3.18 (2.16-4.67)	<0.001	2.92 (1.95-4.37)	<0.001
No	148 (39.3)	1.0		1.0	
WHR					
Normal fatness (<0.85)	172 (44.1)	0.58 (0.40-0.86)	0.006	0.85 (0.54-1.32)	0.4
Central obesity (≥0.85)	87 (57.2)	1.0		1.0	
BMI (kg/m^2^)					
Normal weight (18.5-24.9)	125 (43.1)	0.66 (0.47-0.93)	0.01	0.99 (0.63-1.55)	0.9
Overweight/Obese (≥25.0)	134 (53.2)	1.0		1.0	
Perception of weight					
Underweight/Normal weight	85 (43.1)	0.74 (0.52-1.06)	0.1	0.87 (0.57-1.35)	0.5
Overweight/Obese	174 (50.4)	1.0		1.0	

*Moderate/poor/very poor;

†Adjusted for age, education level, years of service, marital status, job, present known disease, BMI, WHR, and perception of weight status;

BMI=Body mass index;

CI=Confidence interval;

OR=Odds ratio;

WHR=Waist-to-hip ratio

As the study women worked in the health system and, thus, were more familiar with appropriate weight status, we expected a more accurate perception of body fatness. However, their conception was not realistic and healthy in most cases. Besides, considering the prominent role of these women in providing health services, their correct perception would probably lead to improved appropriate perceptions among health service recipients. However, this issue was not investigated.

Overestimation of weight status is a risk factor of unhealthy behaviours in weight control. The high rate of overestimation of weight status among the women in this study showed that the perception pattern of these women might be different from that of the general population; this pattern is more similar to perceptions of the females in some countries, such as the western countries. Nowadays, slimness is considered to be ideal by many women in some countries (30). The women in these countries usually assume their weight to be higher than the actual weight and tend to become slimmer (31,32). Although the use of the foreign media is limited in Iran, it is possible that the perception of the women in this study was affected by the unhealthy models of slimness broadcast by some media, such as the western media. Studies also show that a slim body model is being promoted among the high socioeconomic levels in the developing countries (33). While a big concern of health experts is that, having a higher BMI in the developing countries is suggestive of having a higher socioeconomic level or better sources, which leads to desirability of obesity (34), the tendency towards slimness among some parts of these societies must be regarded.

Further investigations are needed to understand the different aspects of the high rate of misperception in female employees working at the health centres.

Despite the correct perception of body-weight status in most of those with central obesity or BMI-based overweight or obesity in the present study, their perception of health status was illusive; only about 13% of centrally-obese women and 10% of overweight or obese individuals, according to BMI, thought their health status was poor/very poor, and even only 14% of the cases with simultaneous high BMI and WHR reported their health status was poor/very poor. The result of this study did not show a significant relationship between fatness status and perceived health status. The present known disease was the strongest predictor of the perception of health status as women who were afflicted by a disease were more likely to report their perception of health status as poor/very poor when compared with healthy individuals. The frequency of having a known disease was significantly higher in subjects who had central obesity or a high BMI compared to their counterparts (data not shown). However, individuals did not consider their fatness to be a health risk, indicating that they were not aware that excess adiposity could affect their health status.

About half of the women who considered themselves overweight or obese expressed excellent/good health status. Our results are consistent with other studies, indicating that, although overweight people may be aware of their excess weight, they optimistically believe that they are at a lower risk than their peers (35). Obese people also tend to underestimate the morbidity risk (36). However, in a study in the USA, obesity was negatively correlated with self-rated health status in adults, even in the absence of chronic diseases (37) .

### Limitations

The limitations of this study include the following: first, the women's perception of weight was not measured objectively; however, an individual's direct self-perception of his/her weight status is the first step in respecting nutritional and health behaviours. Second, as underweight women were excluded from the study, the findings may be different in those individuals. Third, as the study population consisted of working people, they can be healthier and more aware of their health status than the general population of the same age. Fourth, our study population could be different from the women working at health centres in other cities; hence, the results might not be generalizable to all women working at health centres in the country.

### Conclusions

Despite limitations, this study provided some information about perception of body fatness and health status among an important group of healthcare workers in the society for the first time. Besides, this is among the few studies considering the effect of body-fat distribution on assessing the perception of body-weight and health status.

There was marked discordance between the perceived weight status and actual fatness in women who were of normal weight according to BMI. The individuals’ perception of health status was not associated with their adiposity status. The importance of using body-fat distribution in the perception of weight and health status should be emphasized. According to the findings of the present study, we need appropriate health strategies to improving the knowledge of and developing the insight about appropriate adiposity status and its effect on health status among women working at health centres. The effect of the perception of healthcare workers regarding body-weight status must be considered in weight management interventions.

## ACKNOWLEDGEMENTS

This study was funded and supported by the National Nutrition and Food Technology Research Institute, Grant No. 25.47.6365.

## References

[1] Klauer J, Aronne LJ (2002). Managing overweight and obesity in women. Clin Obstet Gynecol.

[2] Pradhan AD, Skerrett PJ, Manson JE (2002). Obesity diabetes, and coronary risk in women. J Cardiovasc Risk.

[3] Kelishadi R, Alikhani S, Delavari A, Alaedini F, Safaie A, Hojatzadeh E (2008). Obesity and associated lifestyle behaviours in Iran: findings from the First National Non-communicable Disease Risk Factor Surveillance Survey. Public Health Nutr.

[4] Riley NM, Bild DE, Cooper L, Schreiner P, Smith DE, Sorlie P (1998). Relation of self-image to body size and weight loss attempts in black women. Am J Epidemiol.

[5] Flynn KJ, Fitzgibbon M (1998). Body images and obesity risk among black females: a review of the literature. Ann Behav Med.

[6] Park E (2011). Overestimation and underestimation: adolescents’ weight perception in comparison to BMI-based weight status and how it varies across socio-demographic factors. J Sch Health.

[7] Wardle J, Beinart H (1981). Binge eating: a theoretical review. Br J Clin Psychol.

[8] Isomaa R, Isomaa AL, Marttunen M, Kaltiala-Heino R, Björkqvist K (2011). Longitudinal concomitants of incorrect weight perception in female and male adolescents. Body Image.

[9] Omidvar N, Eghtesadi S, Ghazi M, Minaei S, Samareh S (2002). [Body image and its relation to body mass index and food consumption pattern in Tehran adolescents]. Res Med.

[10] Alipoor S, Goodarzi AM, Nezhad MZ, Zaheri L (2009). Analysis of the relationship between physical self-concept and body image dissatisfaction in female students. J Social Sci.

[11] Akiba D (1998). Cultural variations in body esteem: how young adults in Iran and the United States view their own appearances. J Soc Psychol.

[12] Sotoudeh G, Khosravi S, Karbakhsh M, Khajehnasiri F, Khalkhali HR (2008). What women think about their husbands’ opinions might influence women's body image: an explorative study. Indian J Med Sci.

[13] Gregory CO, Blanck HM, Gillespie C, Maynard LM, Serdula MK (2008). Health perceptions and demographic characteristics associated with underassessment of body weight. Obesity (Silver Spring).

[14] Gutiérrez-Fisac JL, García EL, Rodriguez-Artalejo F, Banegas, Guallar-Castillón P (2002). Self-perception of being overweight in Spanish adults. Eur J Clin Nutr.

[15] Linder J, McLaren L, Siou GL, Csizmadi I, Robson PJ (2010). The epidemiology of weight perception: perceived versus self-reported actual weight status among Albertan adults. Can J Public Health.

[16] Hendley Y, Zhao L, Coverson DL, Din-Dzietham R, Morris A, Quyyumi A (2011). Differences in weight perception among blacks and whites. J Womens Health (Larchmt).

[17] World Health Organization (2011). Waist circumference and waist-hip ratio: report of a WHO expert consultaion; Geneva, 8-11 December 2008.

[18] Rahman M, Berenson AB (2010). Self-perception of weight and its association with weight-related behaviors in young reproductive-age women. Obstet Gynecol.

[19] Duncan DT, Wolin KY, Scharoun-Lee M, Ding EL, Warner ET, Bennett GG (2011). Does perception equal reality? Weight misperception in relation to weight-related attitudes and behaviors among overweight and obese US adults. Int J Behav Nutr Phys Act.

[20] Fatimah A, Mdidris M, Romzi M (1995). Perception of bodyweight status among office workers in two gorvernment departments in Kuala Lumpur. Malays J Nutr.

[21] Markey CN, Markey PM, Brich LL (2004). Understanding women's body satisfaction: the role of husbands. Sex Roles.

[22] Millstein RA, Carlson SA, Fulton JE, Galuska DA, Zhang J, Blanck H (2008). Relationships between body size satisfaction and weight control practices among US adults. Medscape J Med.

[23] Lynch E, Liu K, Spring B, Hankinson A, Wei GS, Greenland P (2007). Association of ethnicity and socioeconomic status with judgments of body size the Coronary Artery Risk Development in Young Adults (CARDIA) study. Am J Epidemiol.

[24] Murray S (2006). Is waist-to-hip ratio a better marker of cardiovascular risk than body mass index. CMAJ.

[25] Pischon T, Boeing H, Hoffmann K, Bergmann M, Schulze MB, Overvad K (2008). General and abdominal adiposity and risk of death in Europe. N Engl J Med.

[26] Garrido-Chamorro RP, Sirvent-Belando JE, Gonzalez-Lorenzo M, Martin-Carratala ML, Roche E (2009). Correlation between body mass index and body composition in elite athletes. J Sports Med Phys Fitness.

[27] Jackson AS, Stanforth PR, Gagnon J, Rankinen T, Leon AS, Rao D (2002). The effect of sex, age and race on estimating percentage body fat from body mass index: The Heritage Family Study. Int J Obes Relat Metab Disord.

[28] Blaak E (2001). Gender differences in fat metabolism. Curr Opin Clin Nutr Metab Care.

[29] Inoue M, Toyokawa S, Miyoshi Y, Miyano Y, Suzuki T, Suyama Y (2007). Degree of agreement between weight perception and body mass index of Japanese worker: My Health Up Study. J Occup Health.

[30] Bendixen A, Sørensen TI, Hørder K, Svendsen AJ, Leboeuf-Yde C, Steffensen IE (2007). [The importance of physical activity and body mass index for body satisfaction]. Ugeskr Laeger.

[31] Chang VW, Christakis NA (2003). Self-perception of weight appropriateness in the United States. Am J Prev Med.

[32] Mossavar-Rahmani Y, Pelto GH, Ferris AM, Allen LH (1996). Determinants of body size perceptions and dieting behavior in a multiethnic group of hospital staff women. J Am Diet Assoc.

[33] Khawaja M, Afifi-Soweid RA (2004). Images of body weight among young men and women: evidence from Beirut, Lebanon. J Epidemiol Community Health.

[34] Fernald LC (2009). Perception of body weight: a critical factor in understanding obesity in middle-income countries. J Womens Health (Larchmt).

[35] Renner B, Knoll N, Schwarzer R (2000). Age and body make a difference in optimistic health beliefs and nutrition behaviors. Int J Behav Med.

[36] Falba TA, Busch SH (2005). Survival expectations of the obese: is excess mortality reflected in perceptions. Obes Res.

[37] Okosun IS, Choi S, Matamoros T, Dever GE (2001). Obesity is associated with reduced self-rated general health status: evidence from a representative sample of white, black, and Hispanic Americans. Prev Med.

